# Cognitive Metaphysics

**DOI:** 10.3389/fpsyg.2018.01700

**Published:** 2018-09-11

**Authors:** Lieven Decock

**Affiliations:** Department of Philosophy, Vrije Universiteit Amsterdam, Amsterdam, Netherlands

**Keywords:** conceptual space, identity, metametaphysics, object, Quine, vagueness

## Abstract

In recent years philosophers have been interested in the methodology of metaphysics. Most of these developments are related to formal work in logic or physics, often against the backdrop of the Carnap-Quine debate on ontology. Drawing on Quine’s later work, I argue that a psychological or cognitive perspective on metaphysical topics may be a valuable addition to contemporary metametaphysics. The method is illustrated by means of cognitive studies of the notions “identity,” “vagueness,” and “object” and is compared to other extant metametaphysical positions.

## Introduction

In recent years there has been an outspoken interest in the methodology of metaphysics in the emerging field of metametaphysics. Whereas metaphysicians are interested in the foundations of reality, metametaphysicians are interested in the foundations of metaphysics itself. They want to know whether metaphysical questions are substantive and how to gain metaphysical knowledge, whereby one might consider common sense, conceptual analysis, or quasi-scientific procedures (Chalmers et al., 2009). The interest in metametaphysics is indicative of some problems at the core of the metaphysical project.

A first problem is that many metaphysicians no longer consider the standard formal metaphysical framework adequate. In the 20th century, (analytic) metaphysics, and in particular ontology, became tightly wedded to modern logic (Russell, 1918/2010; Wittgenstein, 1922; Quine, 1948). Quine proposed his famous criterion of ontological commitment “to be is to be the value of a variable,” which states that we are ontologically committed to the entities that are in the range of the existential quantifier in the logical formulations of our best scientific theories. The framework of logic facilitated a formal rigor previously unattainable, but quickly led to new problems and deflationary views. A vivid example is the problem of composition. Physical objects are in general composed of parts, e.g., a watch can be taken apart in several components, which again can be composed of smaller components. It proves to be hard to find general (mereological) principles that express which composed entities are entities in their own right over and above the parts that constitute them. One strategy is to allow unrestricted composition, but it is readily seen that this leads to a deflationary view in which ontological questions become shallow.^1^ For example, Quine (1981, 124), who defends this broad conception of physical objects, explicitly accepts that “[t]here is a physical object part of which is a momentary stage of a silver dollar now in my pocket and the rest of which is a temporal segment of the Eiffel Tower through its third decade.” If every part of space-time contains an object, the concept of objecthood becomes void. Many contemporary philosophers now defend the view that improvements and/or additions to the formal framework should be made so that a clear distinction between substantive and shallow metaphysical questions can be drawn. To this end, typically, extra metaphysical concepts such as “fundamentality” (Sider, 2011) or “grounding” (Fine, 2001) are invoked. Metametaphysics in a narrow sense is concerned with these changes in the standard metaphysical framework.

A second and more serious concern is that many philosophers and scientists regard the methodology of analytic philosophy as deeply flawed. Most results in metaphysics are based on conceptual analysis and on the further formalization of insights gained by conceptual analysis. Several authors (e.g., Ladyman and Ross, 2007; Unger, 2014) argue that an *a priori* analysis of metaphysical concepts cannot yield substantive results. In view of the alleged fickleness of conceptual analysis, it has been argued that metaphysics should become scientific (see Maudlin, 2007; Ross et al., 2013). Most of the proponents of naturalized or scientific metaphysics consider the fundamental theories of physics as the appropriate starting point for metaphysics.

The metaphysics of physics is not without its detractors though. A common complaint is that the added value of metaphysical reflection within physics is not obvious.^2^ Another problem is that traditional metaphysical categories (object, property, cause, time...) are radically transformed within the context of physics. For example, Ladyman and Ross (2007) no longer consider objects or things as the building blocks of external reality, but propose that structures constitute the most fundamental level of reality. On Maudlin’s view (2007), physical laws are fundamental. Important though these insights might be, they leave the layman perplexed, since it is hard to form a conception of the outside world without the more traditional metaphysical concepts. The large gap between the metaphysical concepts in physics and the folk metaphysical concepts leaves room for a descriptive analysis of our folk metaphysical notions. Dennett (1991, 2013) argues that the perspective one should take with regard to metaphysical questions is to view them as questions arising in common situations in daily life. On Dennett’s view, the metaphysician becomes a diplomatic anthropologist who analyzes the use of metaphysical terms in the manifest image, but is not concerned with limning the ultimate structure of reality.^3^ It is surprising that Dennett does not propose to take recourse to the cognitive sciences for this anthropological endeavor, especially in view of the ample use he makes of scientific findings in his theories of consciousness, free will, religion, and cultural evolution.

In this paper, I will explore the prospects of cognitive theories about the metaphysical concepts we use in daily life. I’ll introduce the term cognitive metaphysics^4^ for the study of the basic categories in the human mind that structure the representation of the environment.^5^ Since our understanding of the world is always mediated through these basic categories, reflection and study of them seems unavoidable in a proper study of our metaphysical thought. Cognitive theories of the fundamental categories by which we understand our environment are obviously useful in the cognitive sciences. Also philosophers might profit from this project. As Paul (2010a) argues, insights from the cognitive sciences may lead to refinements of metaphysical intuitions, because subtle unnoticed psychological biases in the conceptual analysis may thus be detected. Osborne (2016) argues that empirical findings will have a destructive impact on common-sense ontology and thus will be of benefit in debunking strategies in metaphysics. I will argue that cognitive studies may undermine particular metaphysical doctrines arrived at by means of conceptual analysis and help us achieve a better understanding of many metaphysical issues and puzzles. Since I consider cognitive studies in general as more reliable than conceptual analysis, my position is slightly more radical than Paul’s. My view is less dismissive than Osborne’s, as I believe that many traditional metaphysical problems are rather easily explained as naturally resulting from the particular make-up of the human cognitive system.

In the next section I start with an overview of Quine’s ontological views and explain that in his later ontological views the basis of a cognitive metaphysics can be traced. In sections “Metaphysical Applications of Conceptual Spaces” and “Objects” I illustrate the possibilities of a cognitive approach in metaphysics by means of three paradigmatic cases: the paradoxes of identity and vagueness in Gärdenfors’s conceptual spaces approach and empirical studies of the notion “object.”^6^ In the fifth section I compare the cognitive approach with logical and physical approaches in metametaphysics. In the last section I discuss several objections that may be raised against the use of empirical findings from the cognitive sciences in metaphysics. I argue that the objections are not fatal, but do highlight some limitations of the approach.

## Quine’s Naturalized Epistemology of Ontology

The current metametaphysical debate can only be understood against the backdrop of Quine’s ontological views. Arguably, the metametaphysical debate was triggered by Azzouni’s (1998) comparison of Quine’s criterion of ontological commitment with possible alternatives, which led to reflection on the role and methodology of metaphysics, and to the question whether metaphysical questions are substantive. Many contemporary philosophers now defend the view that Quine’s views should be amended so that a clear distinction between substantive and shallow metaphysical questions can be made. In this section, I will revisit Quine’s ontological views and point out that in Quine’s later work interesting clues for a cognitive^7^ approach in the metametaphysical debate can be found.

In metametaphysical writings, the Quine-Carnap controversy is often revisited. In his early works, inspired by Russell and the philosophy of logical atomism, Quine defended a substantive theory of ontology. By means of his criterion of ontological commitment “to be is to be the value of a variable” (1948) he provided ontology with a new and rigorous method. Carnap, on the other hand, is known for his deflationary views. In his Vienna Circle period he (Carnap, 1928, 1931) had dealt a serious blow to the metaphysical project by construing metaphysical statements as meaningless. In his response to Quine’s new ontological ideas, he (Carnap, 1950) argued that ontological questions are trivial; either they are internal questions within a chosen logical framework, or they are “meaningless” external questions. Both in Quine’s proposal of an ontological criterion and in Carnap’s critique, it is clear that the logical framework plays a crucial role. Ontology is strongly related to a logical framework, and in Quine’s particular proposal to the role of the existential quantifier in first order logic.

As I have argued in earlier work (Decock, 2002, 2004), Quine’s interest in ontology is far more deeply related to his early work in logic and set theory than is generally assumed. The details of this story need not detain us here,^8^ but the relevant point is that Quine’s ontological ideas are deeply grounded in considerations concerning the existence of abstract objects and the nature of the set-theoretical universe. In “On what there is” (1948) the ontological framework is applied outside the context of mathematics and the criterion of ontological commitment becomes relevant for existential questions regarding ordinary physical objects, such as chairs and tables. However, it is not clear that Quine’s ontological framework is readily applicable outside set theory or mathematics (Decock, 2002). It is not obvious that “our conceptual firsts (…) the middle-sized, middle-distanced objects” (Quine, 1960) can readily be encompassed within Quine’s ontology. Formal disciplines as logic, set theory, and model theory provide neat tools for particular types of ontological questions regarding the realm of abstract objects (often called “Plato’s Heaven”), but are less suited for more mundane ontological questions.

In later years Quine was one of the first to express doubts about the ontological framework he had put forward and became de facto one of the first antirealists in the 1960s. He put forward his deflationary views in “Ontological relativity” (1969), and ontological relativity and the inscrutability of reference became central tenets in his philosophy.^9^ Quine’s new antirealist take on ontology preceded a wave of antirealism, whose most famous heralds were Dummett, Goodman, and Putnam.^10^ Many philosophers have been unhappy with antirealism though. The philosophical motives for antirealism are clear and well-understood and yet antirealism seems to offend the strong belief that there really is a mind-independent world that can be described in a precise language. A clear contemporary expression of this “knee-jerk realism,” and in several ways a return to Quine’s early views, is Sider’s (2011) theory of fundamental truths. Interestingly, in Quine’s later work there is an attempt to mitigate his earlier ontological relativity. Since the mid-eighties, in several articles (Quine, 1984, 1991) and in his latest books (Quine, 1932/1995, 1992), he tried to block the inscrutability of reference by highlighting “immanence,” the fact that we live amid objects we directly experience. Quine addressed the ontological question from an epistemological point of view, for which he coined the term “epistemology of ontology.” Quine’s epistemology is “naturalized” epistemology, the study how human beings as cognitive agents in the world have built theories of the very world around them. The crucial step^11^ in his genetic account of ontology lies in the cognitive process “reification.”^12^ Reification is the process by which human beings start positing the existence of certain types of entities. Thus ontology has become a psychological and sociological construct. Admittedly, this view comes close to certain brands of antirealism, in particular varieties of constructivism, yet it need not entail antirealism. Naturalized epistemology of ontology is in a straightforward way compatible with the existence of an external world that imposes stringent restrictions on the way it can be reified, categorized and described. The starting point is realistic: a human being sitting in an environment being struck by light waves, sound waves, tactile impressions, and reacting to chemical elements in olfactory and taste experience. In the remainder of this paper I want to explore and discuss the scope of a project of naturalized epistemology of ontology and, more generally, of cognitive metaphysics in a present day context.

## Metaphysical Applications of Conceptual Spaces

Gärdenfors’s (2000, 2014) cognitive spaces approach offers a framework in which a cognitive approach in metaphysics can be illustrated by means of several compelling examples. Conceptual spaces are one-dimensional or multidimensional structures, equipped with a metric. Objects are mapped onto points in these spaces and the dimensions of a space correspond to qualities relative to which objects may be compared with each other. Comparisons are made in terms of the metrics defined on the spaces; the closer the objects (or rather, their representations) are in a given conceptual space the more similar^13^ they are in the respect corresponding to the space. To make this less abstract, consider some actual examples of conceptual spaces. One of the simplest examples of a conceptual space is a three-dimensional space with a Euclidean metric defined on it. This space can serve to represent proximity relations between objects in the world: the closer the representations of objects are in the space, the closer the objects are in reality. Another example is auditory space, which is generally taken to be a space with two dimensions, one for pitch, and one for loudness. The closer two “objects” (in this case, sounds) are represented in the space, the more similar they sound. A third example is color space, which arguably is the hitherto best studied conceptual space. Color space is a three-dimensional Euclidean space, with one dimension representing hue – think of the color circle – one dimension representing brightness – which ranges from white to black, through all shades of gray – and one dimension representing saturation – the intensity of the color. More complicated examples of conceptual spaces have been described in the literature, including olfactory space, multidimensional shape spaces, action spaces, and spaces of scientific concepts.^14^ In the conceptual spaces approach, properties and concepts are identified with regions of conceptual spaces. For example, the property of redness is a region of color space and the property of sweetness is a region of taste space. In principle, any set of points in a space counts as a region of that space, but for reasons of cognitive economy only regions with certain characteristics, in particular convexity, are regarded as properties.

Gärdenfors (2014) gives precise characterizations of various ontological categories within the conceptual spaces approach: an “object” is represented by a sequence of points in a set of conceptual spaces, a “property” by a region in a conceptual space, and a “concept” by a sequence of regions in a set of conceptual spaces. More complex structures can account for categories such as “action” or “event.” In the remainder of this section I will present a more elaborate account of the paradoxes of identity and vagueness within the conceptual spaces approach.

### The Paradoxes of Identity

A first application of the conceptual spaces approach concerns the paradoxes of identity, for which Douven and Decock (2010) offer a general account. Well-known paradoxes of identity are the paradox of Theseus’ ship and the statue/lump of bronze paradox. In the paradox of Theseus’ ship, we consider the identity conditions of the ship with which the Greek hero Theseus returned from Crete. Over time, due to wear and tear, one plank at a time gets replaced with another, until eventually all the planks are replaced. The paradox consists in the fact that every replacement by a single plank cannot be believed to alter the identity of the ship, while in the final stage no material of the initial ship is left. We are confronted with the paradox that the ship hasn’t changed and yet cannot be the same. Another paradox is the identity of a statue and the lump of bronze of which the statue is made; they are characterized by different qualities but are composed of the same material.

Douven and Decock argue that the paradoxes can be understood by construing the notion of identity not as the logical notion of identity, but as a slightly different notion related to a cognitive notion involved in identification. There is a welter of psychological research showing that when we compare items with each other, we typically take into account only a subset of the respects in which the object could be found to be similar and that it is a context-dependent matter which subset we take into account. In light of this, the proposal that “identity” is ambiguous and often means “high similarity in all relevant respects” makes it unsurprising that our identity judgments can vary with context. The conceptual spaces approach provides the means to make this proposal more precise.^15^ The relevant respects are the sets of conceptual spaces that play a role in the identity judgment. Similarity between objects is determined by the distances of their locations in the relevant conceptual spaces. Moreover, high similarity is determined by a particular threshold. If two objects are similar above a certain threshold in all the conceptual spaces that are deemed relevant in a particular context, our judgment is that they are identical. This construal heavily draws on concrete practices in the cognitive sciences; e.g., two shades sufficiently close in color space are considered identical when compared under adequate viewing conditions.

On our construal, the paradoxes of identity are based on a confusion of relevant contexts. In contexts in which we are solely interested in the material a statue is made of, e.g., in order to melt them and reuse the bronze, we ought to judge the statue and the lump of bronze as the same. However, if we attend to certain modal properties, such as its disposition to move through shape space when heated above the melting temperature of bronze, the statue and the lump of bronze are judged to be different. Similarly, the paradox of Theseus’ ship has its origin in the respects that are deemed relevant in the identity judgment.^16^ Disambiguating the relevant contexts will dissolve the paradoxes. This first example illustrates that the use of particular theories of the cognitive sciences may shed light on the basic metaphysical concept “identity” and may lead to solutions for ancient philosophical riddles.

### Vagueness and Borderline Cases

A second example concerns vagueness, a topic that has taken center stage in philosophy in the last two decades. Concepts and predicates can be vague.^17^ For most predicates in our language, we can think of “borderline” cases, neither belonging to the predicate’s extension, nor to its complement. E.g., when we consider a reddish-orangish shade of color, we do not consider it to be clearly red nor clearly orange; the shade is borderline orange-red. Vagueness sits badly with the precision the logical apparatus imposes on contemporary metaphysics, as the principle of bivalence forces us to a yes or no answer to the question whether a particular shade is indeed red. Moreover, many proposals that depart from the principle of bivalence, such as many-valued logics, supervaluation techniques, proposals drawing on fuzzy logic, still have difficulties explaining the nature of borderline cases.

Douven et al. (2013) have developed a model of vagueness within the conceptual spaces approach. The geometrical nature of concepts within this approach allows for a straightforward characterization of a borderline case. We start from the simple case in which concepts are determined by a single prototypical point in a conceptual space. In the case of color space, this implies that each basic color category can be represented by means of a single prototypical color shade. Furthermore, we assume that conceptual spaces can be tessellated into regions associated with the concepts by means of a mathematical technique, Voronoi tessellation. The principle behind Voronoi tessellation is easy; each point in the conceptual space belongs to the category of the nearest prototypical point. We readily see that a borderline case of two concepts is a point that lies at an equal distance of their two prototypical points and not closer to any other prototypical point. E.g., a borderline case of the concepts red and orange lies exactly equidistantly from the prototypical points of red and orange. The important step in Douven et al. is the generalization of this proposal by having a region of prototypical points instead of a single prototypical point. It was already observed by Berlin and Kay (1969/1999) that participants in color categorization tasks select different Munsell chips as exemplifying the most prototypical shade of a particular color category; no unique chips are chosen as prototypical colors. If we furthermore consider the superposition of the Voronoi tessellations of all selections of sets of points in which one point is chosen from each prototypical region, we obtain a “collated Voronoi diagram” with thick concept boundaries. In subsequent papers, we have used this proposal to explain metaphysical notions such as graded membership (Decock and Douven, 2014), namely the idea that an object can belong to a certain set to a certain degree. In the “thick” boundary between concepts, membership of a concept continuously grades off from full membership to non-membership. The next step (Douven and Decock, 2017) was to formulate a theory of graded truth, which allows for straightforward solutions to sorites paradoxes. The details of this work are beyond the scope of this article. The important observation is that starting from a particular cognitive theory, we are able to put forward plausible and precise^18^ answers to questions that have vexed metaphysicians since antiquity.

## Objects

We need not confine ourselves to the conceptual spaces approach to find examples of cognitive approaches in metaphysics. Other theories or frameworks within the cognitive sciences might equally well provide insight in the cognitive nature of our basic metaphysical categories.^19^ Two case studies may exemplify how experiments in the cognitive sciences may clarify the metaphysical notion “object.”^20^ A first study, in which the medieval metaphysical notion of *haecceity* is illustrated by means of an experiment with infants between 3 and 6 years old, was carried out by Hood and Bloom (2007). The infants were asked to take their favorite pet toy to the lab. In the course of the experiment, the toy pet is placed in a so-called duplication machine. First the children get to see how a green log of wood or a rubber animal gets duplicated when placed in the machine. Subsequently their pet is placed in the duplication machine. The children chose in large numbers the pet they thought was their original pet, and some children were so afraid that their pet toy was not allowed to enter the machine. It transpires that from a cognitive perspective a mental directness toward a particular object is more important than the bundle of properties of which it is made up. The particularity, the *haecceity*, of the object is deemed more important than the set of its properties. Moreover, the results tie in neatly with other results in the cognitive sciences; e.g., Pylyshyn’s (2007) FINST (“fingers of instantiation”) theory of objects accords a crucial role to (mental) indexicality in the cognitive process of recognizing objects.^21^

A second study concerns the precedence of spatiotemporal continuity over the set of properties. Scholl (2007) offers interesting results on the phenomenon of object persistence in studies using the tunnel effect. An object with a particular set of properties goes through a tunnel that occludes the object and subsequently reappears with changed properties. If the spatiotemporal trajectory is continued as predicted, observers have an outspoken inclination to see it as the same object with changed properties rather than as a new object. However, as soon as a time delay is observed, observers immediately reify two separate objects. This phenomenon is not restricted to human object perception. Similar experiments have been carried out on animals. In a study by Flombaum et al. (2004), rhesus monkeys are confronted with a tunnel experiment in which lemons are transformed into kiwis during the trajectory. It transpires that the monkeys only suspect that two pieces of fruit are used if there is a time delay with regard to the normal trajectory of a lemon going through the tunnel. We can conclude that spatiotemporal continuity and physically plausible temporal trajectories are essential to our category “object.” Moreover, the examples illustrate that the category “object,” arguably one of the most basic categories in metaphysics, can perfectly be studied in the cognitive science.^22^ When confronted with metaphysical questions whether to choose between a conception of object as a four-dimensional worm in space-time^23^ or as bundles of properties, empirical findings may guide us in our metaphysical deliberations.

## Physicalist, Logical, and Cognitive Approaches in Metaphysics

The previous two sections were aimed at illustrating that a cognitive approach to our basic metaphysical categories can be fruitful. In this section I will situate this cognitive research in metaphysics in the broader metaphysical field, and thus enter the field of metametaphysics. The compatibility with a physicalist worldview and the relation with the traditional logical methodology will be clarified.

One of the reasons why cognitive approaches in metaphysics have largely been neglected is because they are reminiscent of the idealistic and phenomenological traditions. In contemporary metaphysics, the claim, central in idealism and phenomenology, that reality is at the fundamental level mental, is almost^24^ anathema. However, this claim can easily be sidestepped. A study of metaphysical categories ingrained in the human mind can readily be combined with a materialistic worldview. The scientific study of cognition we have considered hitherto implicitly assumes a form of materialism. The starting point is a physical observer placed in a physical environment, and cognitive processes are physical processes within the brain (and parts of the body and the environment, according to defenders of embodied and situated cognition). Metaphysical categories such as object, identity, similarity, property, action, event, and metaphysical topics as compositionality and vagueness, are typically invoked in the description of these cognitive processes. I submit that an in depth study and critical analysis of these metaphysical terms may contribute to a better understanding of the cognitive processes.

Whereas cognitive metaphysics seamlessly fits within a physicalist worldview, not all physicalist approaches in metaphysics leave room for cognitive explanations. A case in point is the view that the only basic metaphysical categories are those involved in our fundamental theories in physics.^25^ We need to distinguish cognitive metaphysics from the “metaphysics of physics.” One might point out that the philosophy of physics is not without its problems. At present we have two different fundamental physical theories, relativity theory and quantum mechanics, and an intense search of half a century for a unifying Theory of Everything has not led to important breakthroughs. Moreover, both theories, but in particular quantum mechanics, elude our common ontological intuitions. These caveats notwithstanding, the quest for the ultimate structure of the physical universe has led to remarkable successes. The aim of cognitive metaphysics is different; it is the endeavor to clarify the structure by means of which human beings understand the world. The categories of physics need not coincide with the categories that are fundamental in the cognitive apparatus by means of which human beings (or other animals) understand their environment. The history of philosophy tells us that at some times philosophers have stressed that categories are forms or essences that are inherent in the world, while at other times, it has been claimed that the categories are imposed by the mind on the worldly phenomena. In the light of the scientific developments in physics and the cognitive sciences in the 20th century,^26^ one should avoid conflating the two endeavors and disambiguate cognitive metaphysics from the metaphysics of physics. With regard to the specific examples discussed above, one must conclude that it is misguided to relate the middle-sized middle-distanced objects we experience in our daily environment to the elementary particles in our physical theories and that it is misguided to try to relate the vagueness in our categorization to vagueness at the level of elementary particles (e.g., quantum indeterminacy).

Whereas cognitive metaphysics can be clearly distinguished from the metaphysics of physics, the relation between cognitive metaphysics and metaphysical theories that relate the metaphysical categories to a logical framework is more intricate, because of the intimate relation between theories of cognition and logic. Historically, logic has sprung from an epistemic motivation. Syllogisms are first discussed in Aristotle’s *Organon* and were designed as a guide for valid reasoning. Leibniz proposed to develop a system of signs, a *characteristica universalis*, perfectly representing concepts, so that by means of a method of mechanical manipulations of the signs, a *calculus ratiocinator*, reasoning processes can be carried out. This project is further elaborated in Boole’s (1854) *The Laws of Thought*, and in Frege’s (1879/1969) *Begriffsschrift*, and the titles of these works highlight the relation between logic and cognition. Turing’s (1936) seminal work on the decision problem in logic triggered the development of the modern computer and the project of Artificial Intelligence. These developments again influenced psychologists who started using computer metaphors in their models of human cognition, and in philosophy of mind functionalism became fashionable. This brief sketch suffices to illustrate the intimate relation between logic and the cognitive sciences.

In recent years we have witnessed important shortcomings of the logical paradigm in the cognitive sciences. Psychological experiments in which it is tested to what extent human beings abide by the logical rules when reasoning yield disconcerting results. A recent line of psychological research studies the quick and dirty heuristic rules people really use in reasoning (see, e.g., Gigerenzer, 2010; Kahneman, 2011).^27^ Another problem is that it is not clear how the logical framework is built-in in the brain. Increased knowledge of brain processes has led to a wave of connectionism since the 1980s. Since the 1990s, based on work in robotics, neuroscience, psychology, and philosophy, the idea that cognition is embodied, situated, enactive, and social, has become ever more prominent. Moreover, probabilistic methods (see Oaksford and Chater, 2007) have led to a “new paradigm psychology of reasoning,” (Over, 2009) in which logic plays a less prominent role. We may safely conclude that contemporary cognitive science and logic have grown apart, at least in important respects.

This gap is important from a metametaphysical point of view. If we choose for the neat and precise logical apparatus to address ontological questions, we soon end up with logical tools such as existential quantification, set theory, and model theory. If we want to address modal questions, i.e., questions related to necessity and possibility, we are soon deeply immersed in modal logic, possible world semantics (Kripke, 1980), or a discussion of the Barcan formula (Williamson, 2013). The method is clear and appropriate for a wide range of metaphysical issues. In particular for metaphysical questions in mathematics the logical approach is the most natural methodology.^28^ However, it is less obvious that the logical methodology is well suited for metaphysical questions concerning mundane objects. The precision and bivalence imposed by the logical apparatus are less suited for objects such as chairs and tables. Are chairs and persons really the values of the variables bound by the existential quantifier in our best theories of the world? Some authors have raised the question whether ordinary objects actually exist.^29^ Thomasson (2007) offers a lengthy argument against the deflationist view that ordinary objects do not really exist over and above more fundamental objects (the elementary particles of physics). One should concede that ordinary objects indeed belong in our ontology, but at the metametaphysical level a cognitive metaphysics will provide a better methodology to address metaphysical questions regarding everyday objects.

## But Is Cognitive Metaphysics Still Metaphysics?

Some worries will remain. Whereas few philosophers and even less cognitive scientists would dispute the feasibility of experimental work in the cognitive sciences on topics, such as object perception, object persistence, or vagueness, many traditional philosophers will downplay the relevance of experimental result within metaphysics. In this last section I will address some objections that can be raised. I will not be able to refute all the objections, as some are related to deeply controversial tenets over which no consensus is to be expected soon, but at least the basic assumptions in my replies are generally accepted within mainstream philosophical traditions.

First, it may be objected that science and philosophy are distinct disciplines with different aims and topics, so that science cannot be relevant for metaphysics. This position has been quite influential in the 20th century, but the same goes for the opposite position that philosophy is continuous with science. In recent decades, we have witnessed an outspoken increase in philosophers that invoke empirical results to stave their arguments. Readers interested in philosophical psychology may even find it remarkable that some would doubt the relevance of scientific results in philosophy. However, a more modest version of the objection may be more forceful. If it were the case that the gap between empirical research and the metaphysical questions we are engaged in is so wide that the metaphysical questions are transformed beyond recognition, the prospect of scientific metaphysics may be jeopardized. In a review of Ladyman and Ross (2007) and Dorr (2010) argued that findings in quantum mechanics are so counterintuitive and so remote from the more mundane concerns in traditional metaphysics that they are of limited interest for traditional metaphysicians. This objection is less compelling with regard to empirical findings in the cognitive sciences. The very reason for invoking the cognitive sciences in metaphysics, rather than physics or logic, as intimated in the previous sections, is that one remains closer to mundane metaphysical topics about ordinary objects. The objection that cognitive science is irrelevant for metaphysics is not persuasive in the absence of additional arguments.

A second objection is that the proposal in not concerned with metaphysics but with epistemology. Cognitive science, it is argued, cannot tell us how the world really is, but only how we gain knowledge about how the world is. The objection relies on the widespread belief that there is a sharp distinction between both. Two lines of response are possible.

A first line of response would be to accept the objection but downplay its importance. If we can claim that in typical metaphysical problems, the real point of contention is epistemological rather than metaphysical, the objection loses much of its appeal. In general, metaphysical deflationists will find this claim congenial. For the purpose of illustration, we reconsider the paradox of identity evoked in the example of Theseus’ ship. If we endorse the deflationary view that the content of every region of space-time,^30^ however, discontinuous, is a separate object and identical to itself,^31^ the metaphysical identity question becomes trivial. If we claim that Theseus’ ship is a single object, we thereby imply that there is a single space-time worm whose contents are Theseus’ ship. Replacing a plank will not change the identity of the space-time worm; at most, it can make us wonder whether we have unambiguously picked out one single space-time worm. The interesting question has become epistemological: how do we *identify* Theseus’ ship with a single space-time worm? On this view, we may admit that cognitive metaphysics can be regarded as changing the question from what there is to what we believe there is. Nevertheless, if questions concerning what there is indeed trivial, the change to the question what we believe there is will be the only way to salvage the traditional metaphysical issues. Moreover, the fact that metaphysical questions are epistemological questions in disguise, cannot be a reason to drop the questions altogether. In various important societal contexts (medicine, the arts, international law) deliberations over certain metaphysical questions, in particular identity questions (Is an embryo a human being? Have the Chapman brothers transformed or destroyed the Goya etchings? Which Kuril Islands are parts of Japan?), often turn out to have great practical consequences.

A second response is more direct. The objection is rebutted if we can argue that there is no genuine distinction between metaphysics and epistemology. Several prominent philosophers have indeed elaborated frameworks in which all putative metaphysical questions eventually turn out to be epistemological questions. A clear example is Kant’s treatment of the Aristotle’s ontological categories. Within the Kantian framework, direct access to the world is lost, and Aristotle’s worldly categories have become concepts structuring the human understanding. In Kant’s (1781/1929, B106) table of categories, categories such as existence, quality, modality, etc., were no longer considered as fundamental features of an external reality, but as the constitutive principles describing the way we understand reality. For present purposes, the Kantian framework remains deeply unsatisfactory in one important respect: Kant’s categories are *a priori* and not open to empirical study. However, if we consider the categories as psychological concepts (implemented in the brain) that structure the way humans understand reality, we arrive at a position where cognitive studies become highly relevant in metaphysics. The view that ontology is subservient to epistemology has also been defended in present day philosophy. Quine’s view that our ontology is determined by our best scientific theories and Putnam’s (1981) internal realism are clear cases in point.

A third and related objection is that the proposal involves a vicious circle. One may argue that in a quest for the fundamental features of our understanding of reality, we cannot avoid relying on our cognitive system and hence employ the very mechanisms we are looking for. Though no straightforward rebuttal to the objection is forthcoming, the force of the objection is limited. The quest for fundamental “metaphysical” structures in the human cognitive apparatus is hardly more problematic than the use of cognition in the cognitive sciences, or the use of perception in the study of vision. Nevertheless, the objection does impose a restriction on the ambition of cognitive metaphysics, as it makes clear that no “ultimate” foundations will be found. Some metaphysicians will justifiably complain that this amounts to an unwarranted retreat from the traditional aims of metaphysics. Other philosophers though, most notably Quine, have argued that there is no escaping this circularity. Quine’s (1969) defense^32^ of naturalized epistemology rests on the claim that there is no external point from which reality and/or cognition can be considered.

A fourth objection is that we can no longer attain certainty in metaphysics. Since science is fallible, its application in metaphysics will yield results that can be superseded by later results. Again this objection is not fatal. Many philosophers and nearly all scientists will accept fallibilism in general and will be unsurprised that metaphysics cannot escape from this predicament. The point deserves some further elaboration though. In the examples presented above specific experimental results and theories in the cognitive sciences were mentioned, and most notably the conceptual spaces framework. The analyses concerning identity, vagueness, and objecthood can only be compelling insofar the cognitive theories they invoke have been corroborated.

A fifth objection is that the scope of cognitive metaphysics will probably be confined to particular metaphysical problems that occur in mundane contexts, whereas the basic structures in our cognitive system will offer little guidance in metaphysical questions that rise within theories in theoretical physics or in mathematics. I do not consider it problematic that certain sets of metaphysical questions are to be answered within fundamental theories such as physics and mathematics, whereas mundane metaphysical questions are more appropriately analyzed from a cognitive perspective. There may even be realms where neither the make-up of our cognitive system nor the formal theories and models of a particular science offer sufficient guidance in metaphysical deliberations. In recent years, considerable energy has been spent on the development of ontologies for sciences such as biomedicine, genetics, or geography.^33^ The entities described in these sciences and their mutual relations are not unambiguous and the development of these sciences may profit from “ontological engineering,” the streamlining of structural relations between the entities posited by these sciences. Cognitive metaphysics can be complementary to these other metaphysical approaches.

A sixth objection that immediately follows is that the unity of metaphysics is abandoned. The prospect of a “disunity of metaphysics” seems unappealing, since metaphysics has always been supposed to provide us with the most fundamental structures and categories within the world. This worry is not easily brushed away.^34^ Yet the various metametaphysical positions discussed, i.e., logical approaches, the metaphysics of physics, and cognitive metaphysics, are in different ways a continuation of the metaphysical project started in Ancient Greece. Even if we drop the requirement that the way the world is ordered coincides with the way we conceive it, we may continue the metaphysical tradition and continue the quest for the most basic categories by means of which the world is ordered or, in a project of cognitive metaphysics, by means of which we can understand our environment.

## Author Contributions

The article was written entirely by LD.

## Conflict of Interest Statement

The author declares that the research was conducted in the absence of any commercial or financial relationships that could be construed as a potential conflict of interest.

## Footnotes

1 For a debunking of the folk notion of composition, see Rose and Schaffer (2017), and for a reply, see Korman and Carmichael (2017). A full discussion of the problem of composition within cognitive metaphysics goes beyond the scope of this paper, but it is clear that composition can be studied empirically within the cognitive sciences, see, e.g., Cacchione (2013).

2 This question is discussed in several contributions in Ross et al. (2013). A similar question is also relevant within the metaphysics of mathematics, see Maddy (2007) for a discussion. See also Daly and Liggins (2014) for a balanced discussion.

3 Similar views have been put forward by earlier philosophers; Strawson’s (1959) influential “descriptive metaphysics” is a clear case in point.

4 The term “metaphysics” is not uncommon in the cognitive sciences; article titles include phrases such as “infants’ metaphysics” (Xu, 1996) in developmental psychology or “ape metaphysics” (Mendes et al., 2008) in a study of animal cognition.

5 The position is of course not entirely new, but remarkably few philosophers have explored it until recently. In an otherwise comprehensive introduction to metametaphysics, Tahko (2015, 208) discusses the prospect of naturalized metaphysics: “This [autonomous] type of metaphysics is not interested in listing the various fundamental particles: fermions, bosons, … Rather, it is interested in listing the most basic categories that the fundamental ‘building blocks’ belong to. […] An important part of autonomous metaphysics is to determine how many of these fundamental categories there are.” No suggestion is made that the categories themselves could be naturalized by means of the cognitive sciences. A notable exception is Alvin Goldman, who has in recent decades consistently dealt with various metaphysical topics from a cognitive perspective, see Goldman (1987, 1989, 1992, 2007, 2015).

6 In this paper I mainly focus on ontological examples. Cognitive metaphysics need not be confined to ontology; also other metaphysical themes, such as modality (Goldman, 1992) or conditionals (see, e.g., Douven, 2016a), could profit from a cognitive approach. Some metaphysical topics, in particular causality (e.g., Paul, 2010a), time (e.g., Paul, 2010b), or color (see Chirimuuta, 2015 for a recent overview), have been extensively studied from a cognitive perspective.

7 In view of the development of the cognitive sciences in recent decades, at least one central tenet of Quine’s philosophy, behaviorism, will have to be abandoned. Quine’s views were strongly influenced by Watson’s and Skinner’s behaviorism, which is regarded as an obsolete doctrine by most psychologists.

8 In Quine’s (1932/1995) early work in logic and set theory, his doctoral dissertation, written under the supervision of Whitehead, and the handbook *A System of Logistic* (Quine, 1934), ontology hardly plays a role. In Quine’s logical frameworks the existential quantifier is not even a logical primitive. Quine’s ontological breakthrough can be dated around 1937 (Quine, 1937). In order to avoid Russell’s notorious paradox, he (1937) imposed an “ontological” restriction on the expressions that were said to express sets. It is clear that ontological considerations in set theory were still at the heart of Quine’s (1963) ontological views in his handbook on set theory. At almost every point in the comparison of the various set-theoretic systems, the existential commitments of the axioms that determine which sets exist within a given set theory are meticulously discussed and theory choice is guided by existential considerations such as the existence of a universal set, higher ordinals numbers, or inaccessible cardinal numbers.

9 Quine (1964) first expressed his doubts about ontology in an article discussing the Löwenheim-Skolem theorems. These theorems state that a theory does not uniquely determine an interpretation and hence does not uniquely determine an ontology. The argument was also central in Putnam’s (1980) argument for antirealism.

10 For a reinterpretation of Putnam’s antirealism within cognitive metaphysics, and more in particular within the conceptual spaces approach, see Decock and Douven (2012).

11 Quine presents a speculative account of the genesis of ontology. In his particular account (Quine, 1995), the crucial step lies in the use of focal observation sentences: “The crucial step to reification of ravens can be achieved by just improving our near approximation by changing ‘there’ to ‘it’: ‘Whenever there is a raven, it is a black raven’ … I see this pronominal construction as achieving objective reference.” The details of Quine’s story need not detain us here; recent empirical findings in the cognitive sciences will be more illuminative than Quine’s hypothetical account.

12 Goldman (1987) introduces an alternative term “entification.”

13 Similarity is conceived in a geometrical way. Tversky (1977) proposed an alternative mathematical model of similarity. For a discussion, see Decock and Douven (2009, 2011).

14 For a conceptual space representing action concepts, see Gärdenfors (2007) and Gärdenfors and Warglien (2012); for an olfactory space, see Castro et al. (2013); for shape spaces, see Gärdenfors (2000) and Churchland (2012); for an application of conceptual spaces to scientific concepts, see Gärdenfors and Zenker (2011, 2013).

15 The present account offers a general framework of how conceptual spaces can be put to use. Extra assumptions will be needed in a full account of specific paradoxes of identity. We will need to investigate whether the objects in a specific paradox of identity can indeed be represented by means of a sequence of points from different conceptual spaces, as it has been argued that the representation of more complex or abstracts objects is beyond the scope of the conceptual spaces approach. Moreover, more must be said about the nature of conceptual spaces. A first interpretation of conceptual spaces is to regard them as ‘phenomenal’ spaces, but elsewhere I have argued against this view, see Decock (2006). A second interpretation is to view them as constructs built on the basis of similarity judgments by means of mathematical techniques such as multidimensional scaling, see Clark (1992) for a clear explanation. This interpretation has the drawback that the similarity determined by distances within conceptual spaces implicitly depends on more fundamental similarity judgments. For present purposes, the most appropriate interpretation is to regard conceptual spaces as psychophysical or neural spaces implemented in the human body or brain. It may be beyond the scope of contemporary science to provide a full account of the physiological and neural processes underlying the conceptual spaces, but we may be confident that sufficiently close approximations of the underlying spaces can be discovered.

16 It may be difficult though to spell out all the relevant respects for more complex cases such as this example. Shape space and location in space-time seem highly relevant. We would not consider the ship to remain the same ship if its shape differs too much from the original shape, nor would we consider it to remain the same ship if there are discontinuities in the spatiotemporal path back to the original ship. A crucial difference between the respects in which we deem the ships to be identical and those in which we deem them to be non-identical is whether we attend to the proportion of original material that is left in the ship.

17 In the literature, also the vagueness of objects is discussed. The issue is related to identity of objects as discussed in the previous section.

18 For empirical tests of the model, see Douven (2016b) and Douven et al. (2017).

19 Identity and vagueness have also been studied within other frameworks. Studies on identity or sameness are numerous in developmental psychology, see, e.g., Hochmann et al. (2016) and references therein. Vagueness has been studied in psychology (see, e.g., Hampton, 2007) and in Artificial Intelligence (for an overview see, e.g., van Deemter, 2012).

20 For a broader overview, see Carey (2009), and for a collection containing additional relevant studies, see Hood and Santos (2009). Casati (2005) compares different notions of objects and discusses the possibility of unification.

21 For a longer philosophical discussion, see also Skrzypulec (2018). In this discussion it is pointed out that the view should be further elaborated for fission and fusion cases, i.e., cases in which visual objects split or merge.

22 For a more skeptical voice, see Benovsky (2016).

23 Another question might be how to distinguish objects extended in time from events. For a nice discussion of events based on a cognitive approach, see Goldman (2007).

24 In recent years we have witnessed a modest revival of panpsychism, the position that the mental is an essential constituent of the world. Since the 1930s, this position was absent for many decades in metaphysics. For a brief historical account and an overview of contemporary themes, see Goff et al. (2017).

25 For an outspoken defense of this view, see, e.g., Maudlin (2007): “Metaphysics is ontology. Ontology is the most generic study of what exists. Evidence for what exists, at least in the physical world, is provided solely by empirical research. (…) The metaphysical irreducibles are to be provided by physics – quarks, electrons, and space-time, for example – rather than by ‘epistemic priority’.”

26 Several philosophers in the past have believed in an isomorphism between the structure of world and the structure by means of which we understand the world. The connection of form and matter was central in Aristotle’s hylemorphism; Spinoza and Leibniz believed in a connection or parallelism between things and ideas; in Wittgenstein’s (1922) picture theory, the language of logic is claimed to mirror the structure of the world. The view presented in this paper is not compatible with this assumption.

27 Osborne (2016, 205) argues that the brain uses heuristics that yield imperfect and incomplete information about the objects in the world, e.g., in solving inverse optics problem, i.e., in the reconstruction of a 3D-interpretation from 2D visual input.

28 In foundational studies of mathematics there are contenders for logic and set theory though, viz. category theory and homotopy type theory.

29 In 2005 issue 88(4) of *The Monist* was dedicated to ordinary objects, and various authors defended deflationary views. For a broader overview of philosophical topics related to ordinary object, see Korman (2016).

30 This object can be characterized with mathematical precision by means of the subset of the quadruples of real numbers that are the coordinates of points lying within this region.

31 The proposal is used for illustrative purposes only; this characterization of objects is incompatible with some contemporary theories in physics.

32 For a historical reconstruction of the development of Quine’s naturalized epistemology, see Verhaegh (2017).

33 For an introduction to the field of applied ontology, see Arp et al. (2015). In the construction of artificial ontologies cognitive principles are often expedient, see Carstensen (2011).

34 For the metaphysical categories time and cause, a full discussion of which goes beyond the scope of this paper, it is not even controversial that they should be discussed differently in different scientific context. Causality may be part of the human understanding of the world, as Hume argued and Michotte’s (1946/2017) psychological experiments proved. In physics, the concepts of causality and physical law are closely linked. In the social sciences, in recent years a probabilistic new paradigm of causality has emerged (Pearl, 2000). The physical concept of time has been radically transformed with the discovery of the theory of relativity, and in neurology the problem of the neural implementation of time experience is an important open question. For neither of these two concepts, an integrated single approach is conceivable.
